# Supplementary material for: Development and evaluation of the digital-screen exposure questionnaire (DSEQ) for young children
Source: PLoS One. 2021 Jun 22;16(6):e0253313. doi: 10.1371/journal.pone.0253313 (PMC8219135; doi:10.1371/journal.pone.0253313)
Supplement: S1 Table — (DOCX) [file pone.0253313.s001.docx]

**S1 Table:** Likert-type feedback proforma for assessing the face and content validity of the DSEQ.

| Queries | Grading | | | | |
| --- | --- | --- | --- | --- | --- |
|  | **0** | 1 | 2 | 3 | 4 |
| 1. To what extent do you think that the questionnaire will be useful for researchers dealing with children with excessive screen-time? | **-** | Not at all | Minimally | To certain extent | To large extent |
| 1. To what extent do you think the questionnaire will be useful for the caregivers to report the parents perceptions/ problems related to screen-time? | **-** | Not at all | Minimally | To certain extent | To large extent |
| 1. To what extent do you think the questionnaire covers most patterns of usage considered to be associated with screen-time? | **-** | Not at all | Minimally | To certain extent | To large extent |
| 1. To what extent do you think that the questionnaire measures screen-time comprehensively? | **-** | Not at all | Minimally | To certain extent | To large extent |
| 1. To what extent is the language of the questionnaire appropriate and understandable? (considering the fact that screen-time is common in rural/ illiterate and urban/ literate population) | **-** | Not at all | Minimally | To certain extent | To large extent |
| 1. How will you rate the length of the questionnaire? | Very short | Short | Adequate | Long | Very long |
