# Supplementary material for: Development and evaluation of the digital-screen exposure questionnaire (DSEQ) for young children
Source: PLoS One. 2021 Jun 22;16(6):e0253313. doi: 10.1371/journal.pone.0253313 (PMC8219135; doi:10.1371/journal.pone.0253313)
Supplement: S2 Table — (DOCX) [file pone.0253313.s002.docx]

**S2 Table: Socio-demographic characteristics of participants selected in various phases of the study.**

| Characteristics | Average | Number | Percentage |
| --- | --- | --- | --- |
| **Phase 2: Acculturation, face and content validity (N=30)** | | | |
| Age (years) | 32.3 | - | - |
| *Relation to the child* |  |  |  |
| Father |  | 3 | 1 |
| Mother |  | 21 | 70 |
| Both |  | 6 | 2 |
| *Education* |  |  |  |
| Below masters |  | 12 | 40 |
| Above masters |  | 18 | 60 |
| *Employed* |  |  |  |
| Father |  | 2 | 66.7 |
| Mother |  | 15 | 71.4 |
| *Socio-economic status* |  |  |  |
| Up to middle class |  | 21 | 70 |
| Above middle class |  | 9 | 30 |
| **Phase 3: Pilot study (N=40)** | | | |
| Age of children (years) | 3.3 (2 months) | - | - |
| *Education* |  |  |  |
| Below masters |  | 26 | 65 |
| Above masters |  | 14 | 35 |
| *Employed* |  |  |  |
| Father |  | 0 | 0 |
| Mother |  | 40 | 100 |
| *Socio-economic status* |  |  |  |
| Up to middle class |  | 19 | 47.5 |
| Above middle class |  | 21 | 52.5 |
| *Income of the Interviewee* | 21009.33/- |  |  |
| **Phase 4: Assessing the test-retest reliability (N=30)** | | | |
| Age (years) | 34.2 | - | - |
| *Relation to the child* |  |  |  |
| Father |  | 10 | 33.3 |
| Mother |  | 19 | 63.3 |
| Both |  | 1 |  |
| *Education* |  |  |  |
| Below masters |  | 15 | 50 |
| Above masters |  | 15 | 50 |
| *Employed* |  |  |  |
| Father |  | 10 | 66.7 |
| Mother |  | 11 | 57.8 |
| *Income of the Interviewee* | 47250/- |  |  |
| **Phase 5: Internal consistency of DSEQ (N=400)** | | | |
| **Average age in years** |  |  |  |
| Child | 3.5 | 0.9 |  |
| Father | 30.5 | 4.5 |  |
| Mother | 26.9 | 3.9 |  |
| **Father’s education** |  |  |  |
| Upto middle school | 177 | 44.3 |  |
| High school and beyond | 223 | 55.7 |  |
| **Father’s occupation** |  |  |  |
| Unemployed | 3 | 0.8 |  |
| Unskilled/ semi-skilled/ skilled | 272 | 68 |  |
| Clerical/ farmer/ semi-professional | 73 | 18 |  |
| Professional | 52 | 13 |  |
| **Mother’s education** |  |  |  |
| Upto middle school | 190 | 47.5 |  |
| High school and beyond | 210 | 52.6 |  |
| **Mother’s occupation** |  |  |  |
| Homemaker | 346 | 86.0 |  |
| Working | 54 | 14.0 |  |
| **Socio-economic status (B.G. Prasad classification)** |  |  |  |
| Above middle class | 223 | 55.8 |  |
| Up to middle class | 177 | 44 |  |
