# Supplementary material for: Development and evaluation of the digital-screen exposure questionnaire (DSEQ) for young children
Source: PLoS One. 2021 Jun 22;16(6):e0253313. doi: 10.1371/journal.pone.0253313 (PMC8219135; doi:10.1371/journal.pone.0253313)
Supplement: S1 File — (DOCX) [file pone.0253313.s003.docx]

| Department of Community Medicine and School of Public Health,Postgraduate Institute of Medical Education & Research, PGIMER, Chandigarh, 160012 सामुदायिक चिकित्सा विभाग एंव जन स्वास्थय विद्दालय,  स्नातकोत्तर चिकित्सा शिक्षा एवं अनुसंधान संस्थान, चंडीगढ़, 160012 (भारत) website: <http://pgimer.nic.in/> | 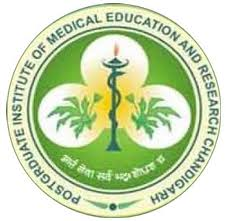 |
| --- | --- |

**PART I. Personal Details (23 items) व्यक्तिगत जानकारी**

| 1. What is the name of the child? बच्चे का नाम क्या है? ________________________________________ | | | | | | |
| --- | --- | --- | --- | --- | --- | --- |
| 1. Who is the primary caregiver of the child? बच्चे की ज्यादातर समय देखभाल करने वाला कौन है?   1) Mother माँ 2) Father पिता 3) Grandfather दादा 4) Grandmother दादी 5) Other अन्य _______________ | | | | | | |
| 1. What is the marital status of the parents? माता-पिता की वैवाहिक स्थिति क्या है?   1) Married विवाहित 2) Widowed/ Widower विधवा/ विधुर 3) Divorced तलाकशुदा 4) Separated अलग  5) Never married कभी शादी नहीं की 6) Others please specify अन्य, कृपया उल्लेख/ वर्णन करें___________ | | | | | | |
| 1. What is the date of birth of the child: बच्चे की जन्मतिथि क्या है ____/____/________ **OR** या   Approximate age (in completed years) of the child बच्चे की आयु लगभग (पूरे किये साल में) कितने साल है ________ | | | | | | |
| 1. Gender of the child: बच्चे का लिंग क्या है: 1) Boy लड़का 2) Girl लड़की 3) Others अन्य | | | | | | |
| 1. Address पता: ____________________________________________________________________ | | | | | | |
| 1. What is your place of residence? आपका घर कहाँ है?   1) Urban शहर 2) Resettlement colony दुबारा बसी हुई कॉलोनी 3) Urbanised village छोटे शहर | | | | | | |
| 1. Total number of rooms in the house: घर में कमरों की कुल संख्या:___________________________ rooms | | | | | | |
| 1. Do you have a garden/ park area in/ near the house where the child can play?   क्या आपके घर के पास या घर में एक बगीचा/ पार्क है जहां बच्चा खेल सकता है? | | | | | Yes (हां)=1 | No (नही)=2 |
| 1. What is your Religion आपका धर्म क्या है? 1) Hindu हिंदू 2) Muslim मुस्लमान 3) Christian ईसाई 4) Sikh सिख 5) Others specify अन्य का उल्लेख/ वर्णन करें __________________________ | | | | | | |
| 1. What is your family type? आपका परिवार किस तरह का है 1) Nuclear family एकल परिवार 2) Joint family संयुक्त परिवार 3) Family with 3 generations (maternal/ paternal cousins) तीन पीढ़ियों वाला परिवार (ममेरे/ चचेरे भाई) | | | | | | |
|  | **13.1 Relation** रिश्ता | **12.2 Age** आयु | **12.3 *Education** शिक्षा | **12.4 ^#^Occupation** व्यवसाय | | **12.5 Income** आय |
| 12.01 | Father पिता |  |  |  | |  |
| 12.02 | Mother माता |  |  |  | |  |
| 12.03 | Head of the family (if other than the father)  परिवार का मुखिया (पिता नहीं है) |  |  |  | |  |
| 12.04 | Elder/ younger sister/ brother छोटे/ बड़े बहन/ भाई |  |  |  | |  |
| 12.05 | Elder/ younger sister/ brother छोटे/ बड़े बहन/ भाई |  |  |  | |  |
| 12.06 | Elder/ Younger sister/ brother छोटे/ बड़े बहन/ भाई |  |  |  | |  |
| **12.3 Education:** *1) Illiterate*: without any formal or non-formal education *2) Primary school literate*: those with non-formal education or those who joined school but did not study beyond 5^th^ standard *3) Middle school* *literate*: those who studies beyond 5^th^ standard but not beyond 8^th^ standard *4) High school literate*: those who have studied till 10^th^ standard *5) Intermediate Diploma*: those who studied till 10^th^ standard and completed a diploma course. Include those with technical education/ diplomas *6) Graduation*: those who have studiedtill graduation *7) Profession or Honors*: those who have studied beyond graduation.  12.3 शिक्षा: 1) अनपढ़: किसी भी औपचारिक या गैर-औपचारिक शिक्षा के बिना 2) प्राथमिक स्कूल साक्षर: गैर-औपचारिक शिक्षा वाले या जो स्कूल में शामिल हुए, लेकिन 5 वीं कक्षा से आगे की पढ़ाई नहीं की है 3) मिडिल स्कूल: वे जिन्होंने 5 वीं कक्षा से आगे की पढ़ाई की लेकिन 8 वीं कक्षा से आगे नहीं 4) हाई स्कूल: 10 वीं कक्षा तक की पढ़ाई करने वाले 5) इंटरमीडिएट डिप्लोमा: उन लोगों को जिन्होंने 10 वीं कक्षा तक पढ़ाई की और डिप्लोमा कोर्स पूरा किया। उन लोगों को शामिल करें जिन्होंने तकनीकी शिक्षा / डिप्लोमा पूरा किया 6) स्नातक: जिन लोगों ने स्नातक की पढ़ाई की है 7) व्यावसायिक या ऑनर्स की डिग्री: वे जो स्नातक से आगे की पढ़ाई कर चुके हैं।  **#12.4 Occupation:** 1) Legislators, senior officials and managers 2) Professionals 3) Technicians and associate professionals 4) Clerks 5) Skilled workers and shop/ market/ sales workers 6)Skilled agricultural & fishery workers 7) Craft and related trade workers: artisans, such as weavers, potters, painters, cobblers, shoe-makers, tailors, etc. 8) Plant & machine operators and assemblers: small scale or cottage industries, industrial/ factory worker, technician such as electricians, masons, plumbers, carpenters, goldsmiths, ironsmiths, those involved in automobile repair works etc., 9) Elementary occupation: includes workers on construction sites 10) Unemployed/ homemaker  # 12.4 पेशा: 1) विधायक, वरिष्ठ अधिकारी और प्रबंधक 2) पेशेवर 3) तकनीशियन और सहयोगी पेशेवर 4) क्लर्क 5) कुशल श्रमिक और दुकान / बाजार / बिक्री कार्यकर्ता 6) कुशल कृषि और मछली पालक 7) शिल्प और संबंधित व्यापार कार्यकर्ता: कारीगर, जैसे बुनकर, कुम्हार, चित्रकार, मोची, जूता बनाने वाले, दर्जी, आदि 8) प्लांट और मशीन चलाने वाले और बनाने/इकटठा करने वाले: छोटे पैमाने पर या कुटीर उद्योग, उद्योगिक / कारखाना कर्मचारी, तकनीशियन जैसे इलेक्ट्रीशियन, राजमिस्त्री, प्लंबर, बढ़ई, सुनार, लौहार, गाड़ियां की मरम्मत कार्यों में शामिल लोग आदि 9) प्राथमिक व्यवसाय: निर्माण स्थलों पर श्रमिक शामिल हैं 10) बेरोजगार / गृहिणी | | | | | | |
| 1. What is the socio-economic status of the family as per Kuppuswamy (refer to the guide)   परिवार की सामाजिक-आर्थिक स्थिति कुप्पुस्वामी (गाइड के संदर्भ में) के अनुसार क्या है ___________________ | | | | | | |
| 1. What is the total number of family members आपके परिवार के लोगों की कुल गिनती _________________ | | | | | | |
| Monthly family income **मासिक आय** 1) ≥78,063/- [12] **2)** 39,033-78062 [10] **3)** 29,200-39,032 [06]  **4)** 19,516-29,199 [04] **5)** 11,708-19,515 [02] **4)** ≤3907 [01] | | | | | | |
| 1. Per capita income of the family परिवार की प्रति व्यक्ति आय_____________________________ | | | | | | |
| 1. Child care facilities or services attended बाल देखभाल सुविधाएं | | | | | | |
| \|  \| 16.02 Number of days per week **उपस्थित दिनों की संख्या** हरेक सप्ताह \| 16.03 Duration/ day (total hours)  **कुल समय** हर **दिन (कुल घंटे)** \| \| --- \| --- \| --- \| \| 16.1 Informal child care (like, family members, nanny) आम बाल देखभाल (उदाहरण के लिए, परिवार के लोग, दाई ) \|  \|  \| \| 16.2 Formal care like नियमानुसार देखभाल जैसे कि (anganwadi आंगनवाड़ी, preschool शिशु स्कूल, play-way प्ले-वे etc.) \|  \|  \| \| 16.3 With the parents at home घर पर माता पिता के साथ \|  \|  \| \| 1. Do you own the following things? क्या आपके घर में यह चीज़ें हैं \| \| \| \|  \| 17.1 Yes हां=1 or या No नहीं=2 \| 17.2 Is the gadget usually placed in the room where the child sleeps/ plays? क्या गैजेट आमतौर पर उस कमरे में रखा जाता है जहां बच्चा घर पर सोता है / खेलता है? \| \| - 1. TV set with cable/ satellite connection टीवी केबल/ डिश कनेक्शन के साथ \|  \|  \| \| 17.02 Computer कम्प्यूटर/ Laptop लैपटॉप \|  \|  \| \| 17.03 Mobile phone without internet मोबाइल फोन \|  \|  \| \| 17.04 Smart phone with internet स्मार्ट फोन \|  \|  \| \| 17.05 Hand-held devices on which video-games can be played (tablet) हाथ में पकड़ने वाला उपकरण जिसपर वीडियो-गेम खेला जा सकता है (नोटबुक) \|  \|  \| \| 17.06 Internet connection (broadband/ WiFi) इंटरनेट कनेक्शन (ब्रॉडबैंड/ वाई-फाई) \|  \| **No answer expected here** \| | | | | | | |

| **PART II. SCREEN TIME EXPOSURE AND Home media environment (27 items) स्क्रीन पर बिताया समय और घर पर मीडिया का वातावरण** |
| --- |

| 1. Types of activities performed by the child, their duration and frequency   बच्चा क्या करता है, कब तक और कितनी बार करता है? | | | 18.1 Watches TV  टीवी देखना | 18.2 Uses a smart phone  स्मार्ट फोन का उपयोग | | 18.3 Uses other gadgets, specify  अन्य गैजेट्स का उपयोग, स्पष्ट करे | | 18.4 Writes/ draws/ colors  लिखना/ चित्र बनाना/ रंग भरना | | 18.5 Reads/ Read by someone  पढ़ना/ किसी द्वारा पढ़ाए जाना | | | 18.6 Others specify कोई भी अन्य | |
| --- | --- | --- | --- | --- | --- | --- | --- | --- | --- | --- | --- | --- | --- | --- |
| 18.01 Frequency of performing the activity in a given week; एक सप्ताह में गतिविधि कितनी बार करता है: 1) Never कभी नहीं 2) Rarely (less than once) शायद कभी (एक बार से भी कम) 3) seldom (1-2 times) शायद ही कभी (1-2 बार), 4) sometimes (3-4 times) कभी-कभी (3-4 बार), 5) Often (5 times or more) अक्सर (5 बार या अधिक | | |  |  | |  | |  | |  | | |  | |
| 18.02 Average duration on working/ school days per day (min) काम करने/ स्कूल वाले दिन प्रतिदिन में बिताया हुआ समय (मिनट में) | | |  |  | |  | |  | |  | | |  | |
| 18.03 Average duration on holidays per day (in min) छुट्टियों वाले दिन कितना समय (*मिनट में)* | | |  |  | |  | |  | |  | | |  | |
| 18.04 Whether the child does the above said activity supervised in a week? एक सप्ताह में बच्चा गतिविधि बड़े की देखरेख में करता है? 1) Never कभी नहीं 2) Rarely (less than once) शायद कभी (एक बार से भी कम) 3) seldom (1-2 times) शायद ही कभी (1-2 बार), 4) sometimes (3-4 times) कभी-कभी (3-4 बार), 5) Often (5 times or more) अक्सर (5 बार या अधिक) | | |  |  | |  | |  | |  | | |  | |
| 1. Which programs/ videos did the child watch yesterday बच्चे ने कल कौन से कार्यक्रम/वीडियो देखे? | | | A | | | B | | C | D | | | | | |
| 1. What was the duration of these programs/ videos इन कार्यक्रमों / वीडियो की अवधि क्या थी? | | |  | | |  | |  |  | | | | | |
| 1. Do you think the placement of TV in the room where the child plays/ sleeps can increases or decreases your child’s screen time? If no, skip to question 24   क्या आपको लगता है कि उस कमरे में, जहाँ बच्चा खेलता/ सोता है, टीवी रखने की जगह आपके बच्चे के स्क्रीन टाइम को बढ़ा या घटा सकता है? यदि नहीं तो प्रश्न संख्या 24 पर चले जाए | | | | | | | | | Yes हां=1 | | | No नही=2 | | |
| 1. If yes, then for how long is the TV switched on in the in the room where the child plays/ sleeps यदि हाँ, तो कितनी देर तक टीवी उस कमरे में चलता रहता है, जहाँ बच्चा खेलता/ सोता है। | | | | | | | | | | | | | | |
| 1. Do you have any rules regarding when, where, what & how to watch digital screen? (If no please skip to question 26)   क्या आपके पास कोई नियम हैं कि कब, कहाँ, क्या और कैसे डिजिटल स्क्रीन देखना है  *(यदि नहीं, तो कृपया प्रश्न 26 पर चले जाए)* | | | | | | | | | | | Yes हां=1 | No नही=2 | | |
| 1. If yes then, what rules do you have for the child at home? यदि हाँ तो घर में बच्चे की डिजिटल स्क्रीन देखने के लिए आपके पास क्या नियम हैं? | | | | | | | | | | |  |  | | |
| 24.1) Only children’s channel allowed केवल बच्चों के चैनल की अनुमति है | | | | | | | | | | |  |  | | |
| 24.2) The child isn’t allowed any media gadget 1h before sleep बच्चे को सोने से 1 घंटा पहले डिजिटल स्क्रीन देखने की अनुमति नहीं है | | | | | | | | | | |  |  | | |
| 24.3) The child is allowed only watch supervised by adults बच्चा केवल बड़ों की देखरेख में देखने की अनुमति है | | | | | | | | | | |  |  | | |
| 24.4) The child isn’t allowed to sit near the TV बच्चे को टीवी के नज़दीक बैठना मना है | | | | | | | | | | |  |  | | |
| 24.5) The child is allowed to watch only for a restricted duration बच्चे को निश्चित समय से अधिक देखने की अनुमति नहीं है __________________ min (*मिनट*) | | | | | | | | | | |  |  | | |
| 24.6) Any other reasons please specify अन्य कृपया उनका उल्लेख / वर्णन करें करे | | | | | | | | | | |  |  | | |
| 1. Caretaker’s media related factors देख-भाल करनेवाले की मीडिया संबंधी बातें | | | | | | | | | | | | | | |
| \|  \| Mother माता \| Father पिता \| \| --- \| --- \| --- \| \| 25.01 Average duration of screen time per day स्क्रीन समय की प्रतिदिन औसत अवधि \|  \|  \| \| 25.02 In a week what is the frequency of media gadget usage **एक सप्ताह में** डिजिटल मीडिया यंत्र उपयोग 1) Never **कभी नहीं** 2) Rarely (less than once) शायद कभी (एक बार से कम) 3) Seldom (1-2 times) **शायद ही कभी** (1-2 **बार**), 4) sometimes (3-4 times) **कभी-कभी** (3-4 **बार**) 5) Often (5 times or more) **अक्सर** (5 बार **या अधिक**) \|  \|  \| \| 25.03 Gadgets used 1) TV टीवी 2) Computer कम्प्यूटर 3) Laptop लैपटॉप 4) Tablet टेबलेट 6) Smart phone स्मार्ट फोन 7) Mobile phone मोबाइल फोन \|  \|  \| \| 25.04 Average time spent with the child at home घर पर बच्चे के साथ बिताया अंदाजन समय \|  \|  \| | | | | | | | | | | | | | | |
| PART III. PHYSICAL ACTIVITY RELATED QUESTIONS (10 items) गतिविधि संबंधित प्रश्न | | | | | | | | | | | | | | |
| 1. 01 Average duration of outside play per day on working/ school days (min) काम करने/ स्कूल के दिन कितना समय घर के भीतर खेलता (मिनट में) |  | 1. What was duration of the following outdoor activities that the child performed yesterday   निम्नलिखित बाहरी कामों की अवधि क्या थी जो बच्चे ने कल की थी | | | | | | | | | | | | |
| 26.02 Average duration on holidays of outside play per day (in min)  छुट्टियों वाले दिन कितना समय घर के भीतर खेलता (*मिनट में)* |  | - 1. Jogged quickly   हलकी सी तेजी से चलना | | | Yes हां=1 | No नही=2 | 27.05 Hopped easy  हलकी से कूदना | | | | | Yes हां=1 | | No नही=2 |
| 26.03 Total duration कुल समय |  | - 1. Tumbled moderately   धीरे से गिरना | | |  |  | 27.06 Hopped moderately  तेजी से कूदना | | | | |  | |  |
|  |  | - 1. Danced नाचना | | |  |  | 27.07 Hopped hard बड़ी तेजी से कूदना | | | | |  | |  |
|  |  | 27.04 Climbed चढ़ना | | |  |  | 27.08 Others अन्य | | | | |  | |  |

**PART IV. MEDIA RELATED BEHAVIORS** मीडिया के प्रति व्यवहार **(15 items)**

| \| 1. In a week, how frequently does the child watch this content on media gadgets at home?   एक सप्ताह में **घर पर मीडिया गैजेट्स पर बच्चा इस सामग्री को कितनी बार देखता है?** \| Never  कभी नहीं=1 \| Rarely (less than once) शायद कभी (एक बार से भी कम)=2 \| Seldom (1-2 times) शायद ही कभी (1-2 बार)=3 \| Sometimes (3-4 times) कभी-कभी (3-4 बार)=4 \| Often (5 times or more) अक्सर  **(5** बार या अधिक**)**=5 \| \| --- \| --- \| --- \| --- \| --- \| --- \| \| 1. The child uses for completing their homework assignments बच्चा कितनी बार स्कूल का काम करने क लिए मीडिया उपकरणों का इस्तेमाल करता है? \|  \|  \|  \|  \|  \| \| 1. The child uses video calling applications to talk to the family/ friends (skype, whatsapp etc.) बच्चा परिवार/ दोस्तों से वीडियो कॉल करने के लिए एप्लीकेशन (स्काइप, व्हाट्सएप, आदि) उपयोग करता है \|  \|  \|  \|  \|  \| \| 1. The child uses for learning poems, rhymes, ABC etc. online बच्चा इन उपकरणों का इस्तेमाल कवितायेँ, अक्षर सीखने के लिए करता है \|  \|  \|  \|  \|  \| \| 1. The child uses to learns maths, numbers, tables online. बच्चा इन उपकरणों का इस्तेमाल गणित, अंक, पहाड़े सीखने के लिए करता है \|  \|  \|  \|  \|  \| \| 1. The child uses to recognize shapes/ sounds/ colors when shown online बच्चा ऑनलाइन दिखाए जाने पर आकार/ आवाज़ / रंगों को पहचानने के लिए करता है \|  \|  \|  \|  \|  \| \| 1. The child learns various sciences online बच्चा विभिन्न विज्ञान से सम्बंधित चीजों को ऑनलाइन सीखने के लिए करता है \|  \|  \|  \|  \|  \| \|  \| Never  कभी नहीं=1 \| Rarely (less than once) शायद कभी (एक बार से भी कम)=2 \| Seldom (1-2 times) शायद ही कभी (1-2 बार)=3 \| Sometimes (3-4 times) कभी-कभी (3-4 बार)=4 \| Often (5 times or more) अक्सर  **(5** बार या अधिक**)**=5 \| \| 1. The child learns to draw, write online बच्चा चित्र बनाना, ऑनलाइन लिखना सीखने के लिए करता है? \|  \|  \|  \|  \|  \| \| 1. The child plays video-games बच्चा **वीडियो-गेम खेल**ता है? \|  \|  \|  \|  \|  \| \| 1. The child uses digital media gadgets to watch stories बच्चा इन उपकरणों का इस्तेमाल कहानियां देखने के लिए करता है \|  \|  \|  \|  \|  \| \| 1. The child to watch adult programs (soap opera, news, sports, movies etc.) बच्चा इन उपकरणों का इस्तेमाल वयस्क कार्यक्रम (ओपेरा,समाचार, खेल आदि) देखने के लिए करता है \|  \|  \|  \|  \|  \| \| 1. The child uses to learns letters, words, vocabulary, language online बच्चा अक्षर, शब्द, शब्दावली, भाषा सीखने के लिए करता है \|  \|  \|  \|  \|  \| \| 1. The child uses to watch random things for enjoyment (music, advertisements, babyTV, click photos etc.) बच्चा मनोरंजन के लिए बिना सोचे समझे देखने के लिए (संगीत, विज्ञापन, बच्चों का टीवी, फोटो खींचने आदि) करता है \|  \|  \|  \|  \|  \| \| 29. Which of the following does your child do as he/ she watches TV? नीचे लिखे में से कौन सा काम आपका बच्चा करता है जब वह टीवी देखता है? \| \| \| \| \| \| \| 29.1 a Talks about the program/ film कार्यक्रम/ फिल्म के बारे में बात करता है \|  \|  \|  \|  \|  \| \| 29.1 b Talks about other things अन्य चीजों के बारे में बात करता है \|  \|  \|  \|  \|  \| \| 29.1 c Talks to the character on the screen स्क्रीन पर आ रहे व्यक्ति/कलाकार से बात करता है \|  \|  \|  \|  \|  \| \| 29.2 Acts out the story/ role-play a character कहानी जैसे काम करता है/ कलाकार के जैसा व्यवहार करता है \|  \|  \|  \|  \|  \| \| 29.3 Sings गाता है \|  \|  \|  \|  \|  \| |
| --- | --- | --- | --- | --- | --- | --- | --- | --- | --- | --- | --- | --- | --- | --- | --- | --- | --- | --- | --- | --- | --- | --- | --- | --- | --- | --- | --- | --- | --- | --- | --- | --- | --- | --- | --- | --- | --- | --- | --- | --- | --- | --- | --- | --- | --- | --- | --- | --- | --- | --- | --- | --- | --- | --- | --- | --- | --- | --- | --- | --- | --- | --- | --- | --- | --- | --- | --- | --- | --- | --- | --- | --- | --- | --- | --- | --- | --- | --- | --- | --- | --- | --- | --- | --- | --- | --- | --- | --- | --- | --- | --- | --- | --- | --- | --- | --- | --- | --- | --- | --- | --- | --- | --- | --- | --- | --- | --- | --- | --- | --- | --- | --- | --- | --- | --- | --- | --- | --- | --- | --- |

**PART V. Media literacy of the parents (11 items)**

**माता-पिता की मीडिया के बारे में जानकारी**

| 1. What do you think are the good things that the child learns from these digital screens?   इन डिजिटल स्क्रीन को देखते समय माता-पिता को क्या लगता है कि बच्चा कौन सी अच्छी चीजों सीखता है? | | |
| --- | --- | --- |
|  | Yes हां=1 | No नही=2 |
| 30.1 The child is learning good habits बच्चा अच्छी आदतें सीख रहा है |  |  |
| 30.2 The child is Increasing his/ her knowledge बच्चा अपना ज्ञान बढ़ा रहा है |  |  |
| 30.3 The child is learning new skills बच्चा नई कला सीखता है |  |  |
| 30.4 It’s good for my child’s growth & development  यह बच्चे की तरक्की और विकास के लिए अच्छा है |  |  |
| 30.5 No positive effects कोई फायदा नहीं |  |  |
| 30.6 Any others specify कोई भी अन्य दिया गया कारण_____________________ |  |  |
| 1. What do you think are the problems when watching these screens excessively?   आपको क्या लगता है कि अधिक समय तक इन उपकरणों को देखने से बच्चे पर इसका बुरा असर हो सकता है? | | |
|  | Yes हां=1 | No नही=2 |
| 31.1 The child starts imitating what he watches  बच्चा जो देखता है उसकी नकल करना शुरू कर देता है |  |  |
| 31.2 The child develops sleep problems  बच्चे को नींद की समस्या हो सकती है |  |  |
| 31.3 The child might start eating unhealthy food  बच्चा अस्वास्थ्यकर भोजन खाना शुरू कर सकता है |  |  |
| 31.4 The child might become aggressive  बच्चा गुस्से वाला बन सकता है |  |  |
| 31.5 The child isolates himself/ herself  बच्चा आस पास की चीजों से अलग खुद को अलग कर लेता है |  |  |
| 31.6 It might Impairs the child’s concentration  यह बच्चे के किसी भी चीज पर ध्यान को कम कर सकता है |  |  |
| 31.7 It might cause behavior problems  यह व्यवहार की समस्याओं का कारण हो सकता है |  |  |
| 31.8 It might impair the child’s eyesight  बच्चे की नज़र को कमज़ोर कर सकता है |  |  |
| 31.9 It’s not good for my child’s growth/development  यह बच्चे की तरक्की/ विकास के लिए अच्छा नहीं है |  |  |
| 31.10 No negative effects कोई नुकसान नहीं |  |  |
| 31.11 Any others specify कोई अन्य का उल्लेख/ वर्णन करें _________________ |  |  |
