# Supplementary material for: Development and evaluation of the digital-screen exposure questionnaire (DSEQ) for young children
Source: PLoS One. 2021 Jun 22;16(6):e0253313. doi: 10.1371/journal.pone.0253313 (PMC8219135; doi:10.1371/journal.pone.0253313)
Supplement: Attachment — Submitted filename: Respone to reviewers comments.docx [file pone.0253313.s005.docx]

PONE-D-20-22585R1
Development and Evaluation of the Digital-Screen Exposure Questionnaire (DSEQ) for Young Children PLOS ONE

| Sr. No. | Reviewers' comments | Response | Page & Line No. |
| --- | --- | --- | --- |
| 1. | The statistics for internal consistency and validity are adequate and appear to partly support the investigators' conclusions. The manuscript is basically descriptive with qualitative and some quantitative conclusions.  However, the investigators make statements such as "Parental intervention, media-literacy, and behaviors were the prime correlates that may directly or indirectly modify the home-media environment, activities performed by the child, media-behaviors, and content watched by their children." This modification possibility is an important consideration. It shows that the authors were incomplete in there analysis. | We agree with the reviewer and have now revised the paragraph as follows:  *This paper reports the five inter-related studies that focused on developing and testing the psychometric properties of a DSEQ and its correlates among children two to five years of age. This study’s results supported the content validity, test-retest reliability, and internal consistency of the DSEQ. The validated DSEQ tool will be useful in measuring DSE and its correlates among young children in India, who have the most significant growing telecom network with approximately 875 million users [26].* | Page 20, line 543-548 |
| 2. | There are 3 tables and 7 supplementary tables with many variables and no effort to coordinate this information in a multivariate format. | We have removed Table S1 on Medical Subject Heading (MeSh) words. MeSH words are given in an already published review by the lead author [Kaur et al (2019)] . This reference [1] is also cited in the text.  *Reference: Kaur N, Gupta M, Malhi P, Grover S. Screen Time in Under-five Children. Indian Pediatr. 2019;56: 773–788. doi:10.1007/s13312-019-1638-8*.  We have now presented the background characteristics of the study participants, in different phases of the study, in a single S3 Table. Supplementary Tables S4, S5, S6, S7 are deleted. We would like to clarify that the study participants in different phases of the study were different, hence we cannot coordinate this information in a multivariate format. In each phase a new set of participants were included.  So now, we have submitted 3 main Tables, 1 Figure, 2 Supplementary Tables and 1 Supplementary File in the manuscript as follows:.  ***Tables***  *Table I: Description of the digital screen exposure questionnaire (DSEQ) items and constructs.*  *Table II: Mean ratings of the content and face validation by experts of the digital screen exposure questionnaire.*  *Table III: Internal consistency and reliability of questions measuring duration, frequency, patterns, and content of screen-time usage among children*  ***Figure***  *Fig 1: Phases of designing the digital-screen exposure questionnaire (DSEQ)*  ***Supplementary Material***  *S1 Table: Likert-type feedback proforma for assessing the face and content validity of the DSEQ.*  *S2 Table: Socio-demographic characteristics of the participants for various phases*  *S1 File: Digital Screen Exposure Questionnaire (DSEQ).* |  |
| 3. | Why was there no consideration of a structure equation modeling (SEM) approach to examine the relationship among underlying structures in the data? | As per González-Montesinos & Backhoff, 2010, the Structural Equation Model (SEM) offers to determine technical procedures and criteria for validation of measurement models under two conditions:   1. Conditional independence, understood as a set of latent factors. For instance, skills, attitudes or perceptions, that influences a group of observed variables measured by the questions that make up a scale. The answers are mutually independent, but it is conditioned and dependent by the latent variable which determines them. 2. Latent factors can be quantified by a dimensional structure based on a Substantive Theory that postulates the existence of psychological constructs that perform a causal influence on people’s responses to a reactive group.   *Reference: Moreno EMO, De Luna EB, Gómez MDCO, López JE. Structural equations model (SEM) of a questionnaire on the evaluation of intercultural secondary education classrooms. Suma Psicol. 2014;21: 107–115. doi:10.1016/s0121-4381(14)70013-x.*  We wish to clarify that in DSEQ, most of the constructs that we have employed were quantitative in nature and were directly observable and it gave direct outcomes rather than latent ones. Therefore, Structural Equation Model was not employed as per the above stated conditions. Also, we would like to further mention that the method and procedure used by us in the present study is widely accepted and used by major researchers in their respective studies for the purpose of development and validation of the study tools [Reference 3 and 29].  This is now included in the discussion.  3. *Lubans DR, Lonsdale C, Plotnikoff RC, Smith J, Dally K, Morgan PJ. Development and evaluation of the Motivation to Limit Screen-time Questionnaire (MLSQ) for adolescents. Prev Med (Baltim). 2013;57: 561–566. doi:10.1016/j.ypmed.2013.07.023*  *29. Grover S, Chakrabarti S, Ghormode D, Dutt A, Kate N, Kulhara P. An Indian adaptation of the involvement evaluation questionnaire: Similarities and differences in assessment of caregiver burden. East Asian Arch Psychiatry. 2011;21: 142–151.* | Page 23  Line 633-638 |
